# Supplementary figures and images for: Close Encounters in a Pediatric Ward: Measuring Face-to-Face Proximity and Mixing Patterns with Wearable Sensors
Source: PLoS One. 2011 Feb 28;6(2):e17144. doi: 10.1371/journal.pone.0017144 (PMC3046133; doi:10.1371/journal.pone.0017144)

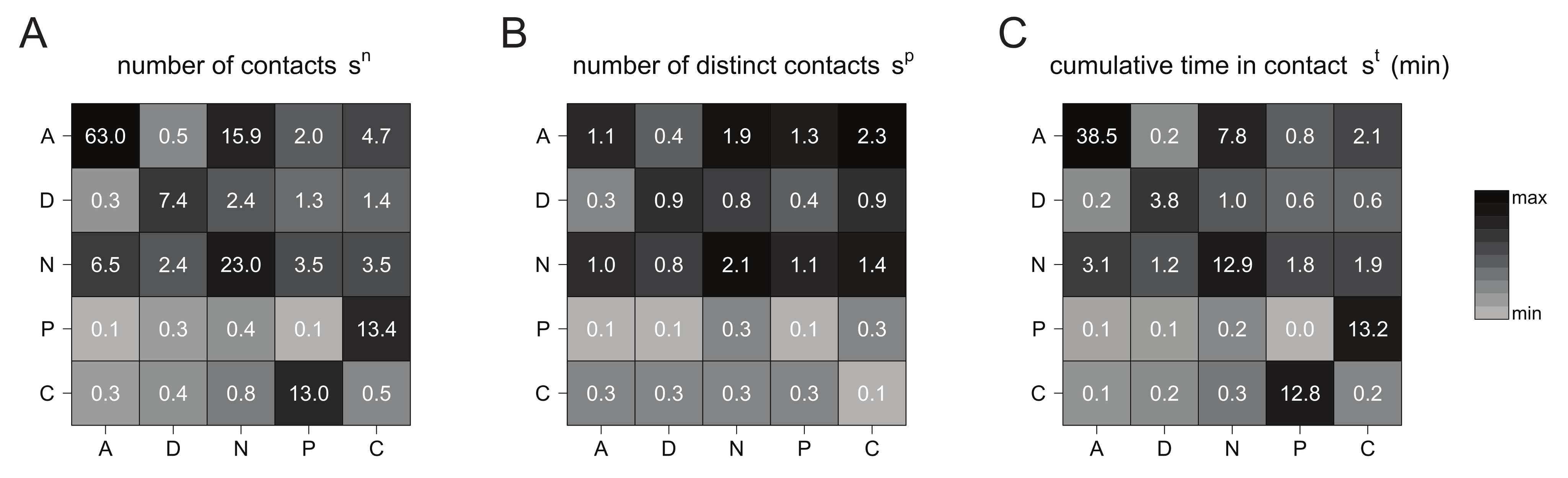

Supplement: Figure S1 — Contact matrices for classes of individuals, with no filtering of RFID badges. The matrices are computed in the same way as those of Figure 5, but no filtering procedure is applied and the data for all RFID badges are retained. Matrices are displayed for the number of contacts (panel A), the number of distinct contacts (panel B), and the cumulative time in contact (panel C). Matrix entries are grayscale-coded according to the median values, with the lightest and darkest shade of gray respectively corresponding to the minimum and maximum value for each matrix. Contact durations are expressed in minutes and normalized to a 24-hour interval. Comparison with Figure 5 shows the robustness of the data with respect to the filtering procedure. (TIF) [file pone.0017144.s001.tif]
