# Supplementary material for: Close Encounters in a Pediatric Ward: Measuring Face-to-Face Proximity and Mixing Patterns with Wearable Sensors
Source: PLoS One. 2011 Feb 28;6(2):e17144. doi: 10.1371/journal.pone.0017144 (PMC3046133; doi:10.1371/journal.pone.0017144)
Supplement: Table S1 — Median number of contacts measured by different roles (D: Physician; N: Nurse; A: Ward assistant; P: Patient; E: Visitor) together with 5th and 95th percentiles (brackets). (DOCX) [file pone.0017144.s002.docx]

|  | A | D | N | P | E |
| --- | --- | --- | --- | --- | --- |
| A | 63.0 [32.1-153.6] | 0.5 [0.3-1.8] | 15.9 [3.6-31.9] | 1.1 [0.1-2.8] | 2.3 [0.5-4.0] |
| D | 0.3 [0.0-0.9] | 7.4 [0.1-31.4] | 2.4 [0.1-8.4] | 0.9 [0.0-2.5] | 0.6 [0.0-3.2] |
| N | 6.5 [2.0-18.0] | 2.4 [0.1-8.8] | 23.0 [4.7-48.6] | 2.0 [0.0-8.7] | 1.9 [0.1-5.3] |
| P | 0.1 [0.0-1.2] | 0.4 [0.0-1.1] | 0.8 [0.0-4.9] | 0.1 [0.0-4.4] | 12.8 [0.0-61.0] |
| E | 0.4 [0.0-2.7] | 0.5 [0.0-1.6] | 0.9 [0.0-5.0] | 15.0 [1.4-63.2] | 0.9 [0.0-7.4] |
