# Supplementary material for: Close Encounters in a Pediatric Ward: Measuring Face-to-Face Proximity and Mixing Patterns with Wearable Sensors
Source: PLoS One. 2011 Feb 28;6(2):e17144. doi: 10.1371/journal.pone.0017144 (PMC3046133; doi:10.1371/journal.pone.0017144)
Supplement: Table S2 — Median number of distinct contacts measured by different roles (D: Physician; N: Nurse; A: Ward assistant; P: Patient; E: Visitor) together with 5th and 95th percentiles (brackets). (DOCX) [file pone.0017144.s003.docx]

|  | A | D | N | P | E |
| --- | --- | --- | --- | --- | --- |
| A | 1.1 [0.8-1.1] | 0.4 [0.2-0.9] | 1.9 [1.0-2.6] | 0.8 [0.1-1.5] | 1.1 [0.3-1.7] |
| D | 0.3 [0.0-0.6] | 0.9 [0.1-1.4] | 0.8 [0.1-1.6] | 0.3 [0.0-1.4] | 0.4 [0.0-1.3] |
| N | 1.0 [0.6-1.1] | 0.8 [0.1-1.1] | 2.1 [1.4-2.4] | 0.6 [0.0-1.5] | 0.9 [0.1-1.4] |
| P | 0.1 [0.0-0.5] | 0.3 [0.0-0.5] | 0.4 [0.0-0.9] | 0.1 [0.0-0.4] | 0.3 [0.0-0.5] |
| E | 0.3 [0.0-0.9] | 0.4 [0.0-0.6] | 0.5 [0.0-1.4] | 0.3 [0.1-0.4] | 0.1 [0.0-0.4] |
