# Supplementary material for: Close Encounters in a Pediatric Ward: Measuring Face-to-Face Proximity and Mixing Patterns with Wearable Sensors
Source: PLoS One. 2011 Feb 28;6(2):e17144. doi: 10.1371/journal.pone.0017144 (PMC3046133; doi:10.1371/journal.pone.0017144)
Supplement: Table S3 — Median cumulative duration (in minutes) of contacts measured by different roles (D: Physician; N: Nurse; A: Ward assistant; P: Patient; E: Visitor) together with 5th and 95th percentiles (brackets). (DOCX) [file pone.0017144.s004.docx]

|  | A | D | N | P | E |
| --- | --- | --- | --- | --- | --- |
| A | 38.5 [15.6-90.5] | 0.2 [0.1-1.1] | 7.8 [1.6-18.0] | 0.4 [0.0-1.4] | 1.0 [0.2-2.0] |
| D | 0.2 [0.0-0.5] | 3.8 [0.0-18.5] | 1.0 [0.0-3.9] | 0.5 [0.0-1.7] | 0.2 [0.0-2.2] |
| N | 3.1 [0.8-9.7] | 1.2 [0.0-4.2] | 12.9 [2.6-28.3] | 0.9 [0.05.2-] | 1.0 [0.0-3.5] |
| P | 0.1 [0.0-0.6] | 0.2 [0.0-1.3] | 0.4 [0.0-3.2] | 0.0 [0.0-3.8] | 11.3 [0.0-64.6] |
| E | 0.2 [0.0-1.2] | 0.3 [0.0-1.0] | 0.5 [0.0-2.9] | 15.3 [0.0-64.9] | 0.3 [0.0-4.5] |
